# Genetic Profiling of Primary Versus Metastatic Ewing Sarcoma for Therapeutic Target Identification

**DOI:** 10.3390/life16060901

**Published:** 2026-05-27

**Authors:** Carly Mitchell, Sarah Voskamp, Eddie Geagea, Deepti Anand, Jennifer Nelson, John Lovejoy

**Affiliations:** 1 University of Central Florida College of Medicine, Orlando, FL 32827, USAsa298761@ucf.edu (S.V.);; 2 Department of Cardiovascular Services, Nemours Children’s Health, Orlando, FL 32827, USA; 3 Department of Orthopedics, Nemours Children’s Health, Orlando, FL 32827, USA; 4 Department of Research, Nemours Children’s Health, Orlando, FL 32827, USA

**Keywords:** Ewing sarcoma, genetics, biomarkers, metastasis, therapeutics

## Abstract

Ewing sarcoma (ES) is a bone malignancy primarily, well known by its t(11;22)(q24;q12) chromosomal translocation. Despite high initial treatment success, ES frequently recurs, likely due to micrometastatic disease present at diagnosis but undetected during primary treatment. This study aims to characterize transcriptomic differences between primary and metastatic ES to identify genes and pathways associated with the metastatic phenotype. Using the Search Tag Analyze Resource for National Center for Biotechnology Information’s Gene Expression Omnibus, seven independent gene expression series were identified, yielding 37 metastatic and 82 primary ES tumor samples. Differentially expressed genes were defined using a significance threshold of *p* < 0.05 and absolute experimental log ratio > 0.1 and were analyzed using Ingenuity Pathway Analysis. This integrative transcriptomic analysis identified 753 significant molecules. Metastatic ES was characterized by upregulation of lung-associated surfactant proteins and secretoglobin family members, along with downregulation of genes involved in extracellular matrix organization. Additional genes of interest included *SLC6A14*, *CXCL14*, and *TBX3*, which have been implicated in tumor progression in other malignancies. These findings provide a computationally derived molecular profile associated with metastatic ES and highlight candidate genes and pathways that warrant further validation. This integrative approach offers a framework for future studies focused on understanding metastatic biology in rare pediatric cancers.

## 1. Introduction

Ewing sarcoma (ES) is a bone tumor composed of small, round cells that primarily manifests as a primary malignancy in the pelvis or lower extremities in pediatric patients [1]. It is the second most frequent bone tumor in the pediatric population [1]. Demographically, white and non-Hispanic patients are at a higher risk of developing ES than their black, Asian, or Hispanic counterparts [2,3]. Male sex is a poor demographic prognostic factor. Tumor size of 8 cm or less, appendicular lesion, and localized disease are positive characteristic prognostic factors [2]. The hallmark transfusion of this malignancy involves the Ewing sarcoma break point region 1 and Friend leukemia virus integration 1 (*EWS-FLI1*) or ETS transcription factor ERG (*EWS-ERG*) [4].

Diagnosis of ES is preferably made by a sarcoma reference center, or the pathology should at least be confirmed by one of these centers. Biopsies are necessary for diagnosis, as imaging can be like other kinds of malignancies or non-malignant pathologies such as osteomyelitis [5]. Clinical symptoms can be similar among all these pathologies, making clinical diagnosis without thorough imaging and biopsy virtually impossible. Other limitations of the diagnostic process include unknown relevance of tumor seeding along the biopsy tract, with studies comparing resection of the tract during the surgical portion of the treatment to have unknown clinical significance currently. Further limitations include special care not to disturb neurovascular structures during biopsies, as these disturbances also have unknown clinical significance [6]. Typical treatment involves three stages: neoadjuvant chemotherapy composed of vincristine, adriamycin, and cyclophosphamide alternating with ifosfamide and etoposide, surgical resection that may be combined with radiation, and finally a second regimen of systemic chemotherapy [4,7]. It is hypothesized that patients have micrometasteses at the time of diagnosis, requiring both local measures such as resection and systemic measures such as chemotherapy [4]. One point of evidence for this theory is that prior to supplemental chemotherapy, ES was treated with local measures only, with almost all patients developing recurrence or metastasis after treatment [4].

The key genetic characteristic of ES being the translocation between chromosomes 11 and 22 (t(11;22)(q24;q12)) [8] is thought to contribute to its genetic potential in several ways. One proposed hypothesis is that ES cells display increased levels of R-loops and dysregulation to the machinery that repairs transcription errors due to *BRCA1* DNA repair and elongation changes [9]. Additionally, malignant potential may be due to lack of inhibition at the G2-M cell cycle arrest point, as therapeutics that inhibit the ES breakpoint region 1 (*EWSR1*) gene and C-terminal of the *FLI1* gene fusion product seem to reintroduce this cell checkpoint [10].

This study is hypothesis-generating and investigates the differentially expressed genes in metastatic ES compared to primary ES, in order to determine genetic aberrations, which could possibly be investigated as biomarkers or therapeutic targets for metastatic disease in further studies.

## 2. Materials and Methods

The National Center for Biotechnology Information, located in Bethesda, MD, USA, maintains the Gene Expression Omnibus (GEO) is a public repository of functional genomics data from prior studies [10]. The Search, Tag, Analyze Resource for GEO (STARGEO) provides a method for identifying and utilizing relevant samples stored in GEO, as well as conducting an integrative transcriptomic analysis to compare functional genomics data between case and control groups. In the current study, the case samples are those from ES metastasis, and the control samples are sourced from primary ES tumors. Series containing samples sourced from cell lines, series lacking both metastatic and primary samples, samples of primitive neuroectodermal tumors, and samples that had been treated or underwent genetic alterations that may alter the integrity of the genomic expression data were excluded from the study. Using STARGEO, seven series were identified that met the criteria (Table 1) and contained both metastatic and primary ES samples, yielding 37 metastatic ES tumor samples and 82 primary ES tumor samples.

Analysis in STARGEO was executed via fixed and random effects models to generate a meta-*p*-value representative of the significance of differential expression between genes expressed in both primary and metastatic ES. STARGEO utilizes a standard meta-analysis method for combining multiple microarray studies described by Choi et al. [16]. Inverse variance weighting and Der Simonian–Laird estimates are utilized within the STARGEO platform to account for inter-study variability when combining datasets [17]. Overall, 22,172 genes were extracted from the random effects model of the samples’ functional genomics data and compared between metastatic and primary ES. These genes were restricted to *p* < 0.05 and an absolute experimental log ratio of ˃0.1 for further investigation in Ingenuity Pathway Analysis (IPA). The experimental log ratio is calculated within STARGEO as log_2_(fold change) and can be interpreted as the magnitude and direction of change in the expression between metastatic and primary ES tumor samples. This step yielded 753 molecules for IPA investigation, of which 453 were upregulated and 300 were downregulated. All microarray probe sites are mapped to Entrez gene identifiers. To strengthen statistical rigor, an independent false discovery rate (FDR) correction (Benjamini-Hochberg) was retroactively applied as a sensitivity analysis. IPA is a bioinformatics software that interprets genomics data and provides insight into the biological significance of the findings (Qiagen Inc., https://digitalinsights.qiagen.com/IPA, 29 September 2024, Fall 2024, Redwood City, CA, USA). There are many functions within this tool, but most notably, IPA maps differentially expressed genes in the dataset onto known canonical pathways to generate predictive analyses, to predict upstream regulators and downstream biological effects based on differentially expressed genes, and to enable identification of potential therapeutic targets and biomarkers of disease. Pathway predictions in IPA are based on an activation z-score algorithm designed to reduce the impact of statistical outliers as described by Krämer et al. [18]. The *p*-value of overlap reported within IPA is calculated via a right-tailed Fisher’s Exact Test with a Benjamini–Hochberg correction to control for false discover rate when combining a large number of genes.

## 3. Results

### 3.1. Gene Candidates

**Figure 1:**
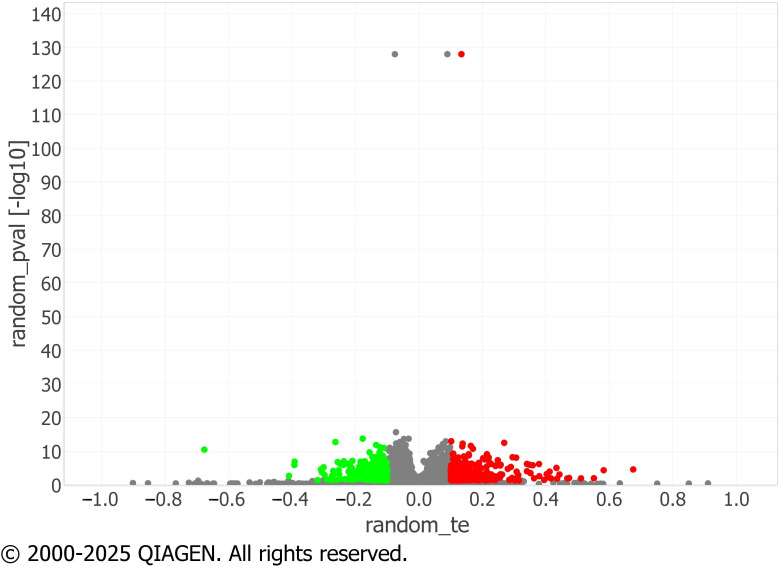
Volcano plot of differential expression of metastatic ES genes compared to primary ES genes. Green circles illustrate upregulated genes, red circles illustrate downregulated genes, and gray circles illustrate genes that did not meet one or both of the required statistical thresholds. Figures generated via QIAGEN Ingenuity Pathway Analysis (IPA) with permission from QIAGEN Silicon Valley.

Of the 753 differentially expressed genes identified between metastatic and primary ES samples, 453 were upregulated, and 300 were downregulated in metastatic ES. Figure 1 demonstrates the genes in a volcano plot distribution. The downregulation of genes in this analysis suggests they are expressed in primary ES tumors and less in metastatic tumors. Table 2 displays the top 10 upregulated and downregulated genes by experimental log ratio in metastatic ES. Of the top 10 upregulated genes, four remained significant with the FDR correction applied (*ZNG1E*, *SFTA3*, *ADH1B*, *SLC6A14*). Of the top 10 downregulated genes, eight remained significant with FDR correction (*SPDYE15*, *OGN*, *CXCL14*, *COMP*, *ENPP1*, *COL14A1*, *MYL1*, *COL12A1*). The FDR corrected *p*-value is included in Table 2. IPA determines the top gene candidates according to the experimental log ratio, which indicates the proportion of change from primary ES on a logarithmic scale. Table S1 supplies a complete list of all genes included for analysis with corresponding log ratios. Table S2 supplies the complete list of significant genes following FDR correction.

### 3.2. Top Canonical Pathways

**Figure 2:**
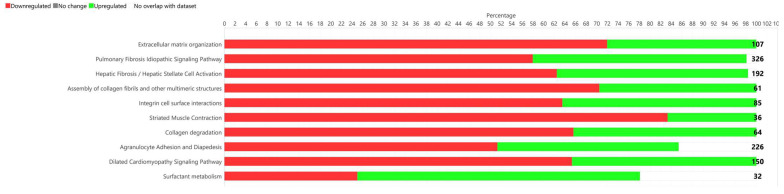
Top 10 canonical pathways associated with metastatic ES versus primary ES. Green bars represent upregulated genes, and red bars represent downregulated genes. Figures generated via QIAGEN Ingenuity Pathway Analysis (IPA) with permission from QIAGEN Silicon Valley.

The top predicted canonical pathways, ranked according to the −log(*p*-value), are extracellular matrix organization (z-score −3.000), pulmonary fibrosis idiopathic signaling pathway (−1.512), hepatic fibrosis/hepatic stellate cell activation (unable to calculate z-score), assembly of collagen fibrils and other multimeric structures (−3.207), and integrin cell surface interactions (−2.324). These canonical pathways, except for hepatic fibrosis/hepatic stellate cell activation, were predicted to be inhibited based on differential gene expression. Collagen type XII alpha 1 chain (*COL12A1*) is a top-downregulated gene included in the canonical pathways of pulmonary fibrosis, hepatic fibrosis, and assembly of collagen fibrils. Cartilage oligomeric matrix protein (*COMP*) is another downregulated gene included in both the extracellular matrix (ECM) formation and integrin cell surface interaction pathways. Figure 2 lists the top 10 canonical pathways.

### 3.3. Upstream Regulators and Causal Networks

Upstream regulators are molecules whose activation or inhibition is predicted to contribute to the observed differential expression in the dataset. By utilizing the *p*-value of overlap derived from right-tailed Fisher’s Exact test *p*-value, the top upstream regulators were ranked. The predicted activation state is determined according to the correlation between the direction of change and the consistency with the literature-predicted direction of change. Similarly, causal networks are molecules predicted to contribute to the observed differential expression of genes in the dataset and are constructed in the same manner as upstream regulators. However, causal networks have a larger degree of freedom and permit indirect relationships between regulators and target genes, whereas upstream regulators enforce direct relationships only. Causal networks also are ranked according to the *p*-value of overlap, thus those identified as the top causal networks have the most significant *p*-value of overlap.

**Figure 3:**
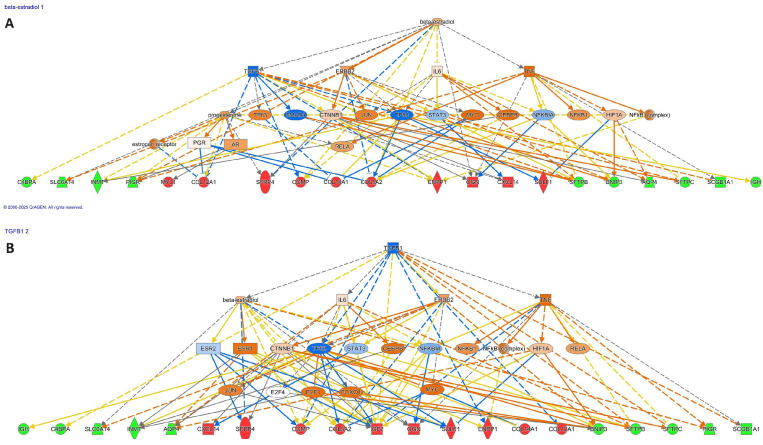
(**A**) Upstream regulator beta-estradiol. Predicted upregulation of beta-estradiol, as described in the Discussion; (**B**) Upstream regulator *TGFB1*. Predicted downregulation of *TGFB1* contributes to the observed increased expression of gene candidates *SFTPC* and *SFTPB*. Figures generated via QIAGEN Ingenuity Pathway Analysis (IPA) with permission from QIAGEN Silicon Valley. The figures demonstrate the predicted downstream alterations due to variation in the expression of the upstream regulator in metastatic versus primary ES. Orange shape is predicted activation, blue shape is predicted inhibition, green shape is increased expression, red shape is decreased expression. Solid line and arrow is direct effect and dashed line and arrow is indirect effect. Orange line is leads to activation, blue line is leads to inhibition, and grey line is effect is not predicted.

The top upstream regulators predicted to contribute to the observed differential gene expression in metastatic ES compared to a primary ES tumor are beta-estradiol (z-score 0.714), transforming growth factor beta 1 (*TGFB1*) (−3.422), dexamethasone (−0.023), T-box transcription factor 3 (*TBX3*) (1.665), and Erb-b2 receptor tyrosine kinase (*ERBB2*) (0.920). *TGFB1* also shows decreased expression in the dataset with an experimental log ratio of −0.036, consistent with the predicted inhibited activation state. Figure 3 displays the downstream effects of beta-estradiol and *TGFB1*, respectively, with upregulated and downregulated genes influenced by *TGFB1*’s inhibition overlaid.

**Figure 4:**
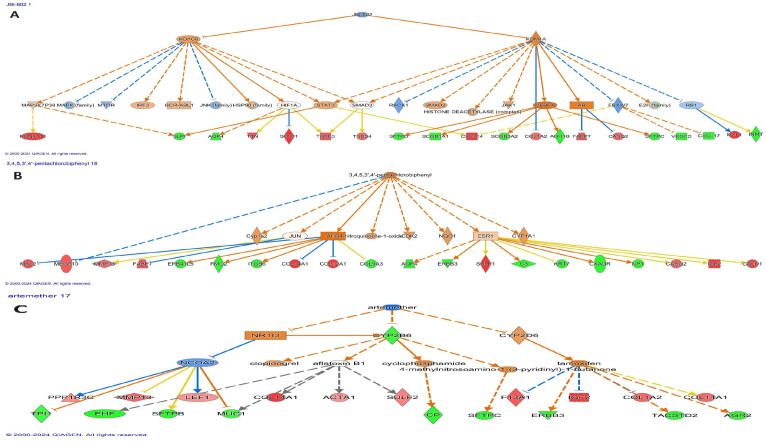
(**A**) Top causal network JBI-802; (**B**) most activated causal network 3,4,5,3′,4′-pentachlorobiphenyl; (**C**) most inhibited causal network artemether. Figures generated via QIAGEN Ingenuity Pathway Analysis (IPA) with permission from QIAGEN Silicon Valley. The figures demonstrate the predicted downstream alterations due to variation in the expression of the upstream regulator in metastatic versus primary ES. Orange shape is predicted activation, blue shape is predicted inhibition, green shape is increased expression, red shape is decreased expression. Solid line and arrow is direct effect and dashed line and arrow is indirect effect. Orange line is leads to activation, blue line is leads to inhibition, and grey line is effect is not predicted.

The top predicted causal networks ranked by the *p*-value of overlap are the chemical drug JBI-802 (z-score: −0.972), the transcription regulator *TBX3* (0.407), the enzyme cytochrome P450 family 2 subfamily B member 6 (*CYP2B6*) (1.497), the transcription regulator Jumonji and AT-rich interaction domain containing 2 (*JARID2*) (−0.786), and the enzyme lysine demethylase 1A (*KDM1A*) (0.641). Despite a significant *p*-value of overlap, none of the top causal networks have a predicted activation state. The most activated causal network, determined by the most positive activation z-score, is the chemical toxicant 3,4,5,3′,4′-pentachlorobiphenyl with a z-score of 4.419 and a *p*-value of overlap 2.85 × 10^−25^. The most inhibited causal network, determined by the most negative z-score, is the chemical drug artemether with a z-score of −4.588 and a *p*-value of overlap 2.48 × 10^−20^. Figure 4 shows the downstream effects of JBI-802, 3,4,5,3′,4′-pentachlorobiphenyl, and artemether, respectively.

### 3.4. Diseases and Biological Functions

The most activated diseases and biological functions predicted to be associated with metastatic ES are transcription of DNA (z-score 2.723), transcription of RNA (2.555), differentiation of epithelial tissue (2.408), activation of endogenous DNA (2.363), and differentiation of epithelial cells (2.317). Interestingly, the most inhibited disease and biological function predicted by IPA was the invasion of cancer cells, with a z-score of −2.844. IPA predicts inhibition of a disease or function if the observed directional change in expression is inconsistent with prior literature.

## 4. Discussion

Prior work describing ES genetic biomarkers and therapeutic treatments has investigated the potential efficacy of insulin-like growth factor 1 (IGF-1R) antibody treatment as it relates to the PI3K/AKT/mTOR pathway [19], in addition to the p16 INK4a mutation’s role in ES pathogenesis [20]. It has been found that the C-X-C motif chemokine receptor 4 (*CXCR4*) gene is associated with a poorer prognosis in bone and soft tissue sarcomas [21]. Interestingly, our data suggests that this gene is downregulated in metastatic ES. One systematic review of ES molecular and biological markers investigated several molecules and how they relate to prognosis, but not to metastasis, and these molecules are non-overlapping with our top genes of interest [22]. A study by Yin et al. reveals one overlapping gene in our dataset, secretoglobin (*SCGB*), which was found to be upregulated in ES when compared to nonmalignant tissue, with the top upregulated physiological processes being muscle contraction and morphogenesis, cell mitotic nuclear division and microtubule-based process, cell adhesion, heat generation process, and gluconeogenesis [23]. Most of the genes identified in our study, including the following detailed analysis, have not yet been investigated in an integrative transcriptomic analysis as they relate to ES pathogenesis or to ES metastatic potential. The following upregulated and downregulated genes demonstrated observed expression changes between metastatic and primary tumor cell samples. As a hypothesis-generating study, these represent genes with potential clinical significance and warrant further investigation to elucidate the definitive biological role in metastatic ES tumor pathogenesis. The proposed hypothesis requires experimentally validated biological studies.

### 4.1. Upregulated Genes

#### 4.1.1. Zn-Regulated GTPase Metalloprotein Activator 1E (*ZNG1E*)

In the current analysis, *ZNG1E* was the most upregulated gene in metastatic ES and encodes a protein implicated in maintaining intracellular zinc homeostasis [24]. In addition to the primary *EWSR1* fusion with *FLI1* (t(11;22)), other fusion sites, including non-ETS-fused round cell sarcomas (NERS), make up <5% of ES cases [25]. Some of these anomalies include fusion of the *EWSR1* gene to the zinc-finger DNA-binding domain, interestingly, upregulating expression of *ZNG1E*, and the subsequent creation of Zn-finger proteins. This suggests a higher demand for Zn utilization and may explain the necessity for *ZNG1E* upregulation in metastatic non-ETS-fused round cell sarcomas [26]. Additionally, non-ETS-fused round cell sarcomas are more likely to be extra-skeletal, suggesting the upregulation of *ZNG1E* in metastatic ES allows it to thrive in extra-skeletal environments when compared to non-metastatic ES [26].

#### 4.1.2. Lung Surfactant Proteins: Surfactant-Associated Protein 3 (*SFTA3*), Surfactant Protein C (*SFTPC*), Surfactant Protein B (*SFTPB*)

*SFTA3*, *SFTPC*, and *SFTPB* are lung surfactant proteins contributing to the production of pulmonary surfactants and reduction in alveolar surface tension. This function may contribute to the predilection of metastatic ES in the pulmonary environment [27]. However, this finding also may be due to surrounding lung tissue in the metastatic tumor samples sourced from pulmonary sites.

It has been shown that inflammatory cytokines interleukin 1 B (IL-1B) and interleukin 23 (IL-23) inhibit expression of *SFTA3* [27], suggesting a possible important therapeutic approach for metastatic ES.

The pre-cleavage form of *SFTPB*, pro-*SFTPB*, has been studied as an upregulated circulating biomarker for both lung cancer [28] and osteosarcoma [29]. Interestingly, Feng et al. also found that osteosarcoma patients with high serum pro-*SFTPB* expression levels had shorter survival times with statistical significance. They also were significantly more likely to have metastasis and more severe tumor clinicopathological categorizations than patients with low serum pro-*SFTPB* expression levels [29]. These findings suggest that *SFTPB* and pro-*SFTPB* have the potential to be used as key circulating biomarkers for ES metastasis or progression of metastatic ES during treatment.

#### 4.1.3. Solute Carrier Family 6 Member (*SLC6A14*)

*SLC6A14* has been widely implicated in malignancies, so its involvement in metastatic ES is not unsurprising. Some of the proposed mechanisms include upregulating glutaminolysis and serine-glycine-one-carbon pathways, which are both involved in necessary metabolic pathways for the growth of cancer cells [30]. One study that utilizes murine models shows that deletion of *SLC6A14* inhibits tumor proliferation [31]. Future studies validating the role of *SLC6A14* in metastatic and primary ES are indicated.

#### 4.1.4. Secretoglobin Family 3A Member 2 (*SCGB3A2*)

Several protein products from the secretoglobin gene family serve as biomarkers of malignancy, while others are believed to be tumor suppressor genes. *SCGB3A2* is thought to be upregulated by thyroid transcription factor 1 (TTF-1; NKX2-1), which is a homeodomain transcription factor necessary for lung, thyroid, and forebrain development. Utilizing *SCGB3A2* as a serum biomarker for either the presence of ES or tracking the progression of the tumor throughout the treatment course should be investigated further, as expression of *SCGB3A2* was found in 21 of the 37 metastatic ES and 69 of the 82 primary ES samples [32]. It also is suggested that interleukin (IL-10), an anti-inflammatory cytokine, upregulates *SCGB3A2* messenger RNA (mRNA) expression [33].

### 4.2. Downregulated Genes

#### 4.2.1. Chemokine (C-X-C) Ligand 14 (*CXCL14*)

This gene’s protein is a cytokine involved in immunoregulatory and inflammatory processes. Some studies suggest it is an important tumor suppressor gene that is most expressed in epithelial cells [34]. Thus, it can be hypothesized that *CXCL14* may exhibit similar tumor suppressor properties in metastatic ES, making downregulation a possible contributor to metastatic growth. Epidermal growth factor is one growth factor suggested to suppress *CXCL14* via the ERK/AKT/mTOR pathway, leading to decreased apoptosis and increased cell proliferation [35].

#### 4.2.2. Cartilage Oligomeric Matrix Protein (*COMP*)

*COMP* is a non-collagenous ECM glycoprotein secreted in a variety of tissues, including cartilage, meniscus, ligaments, tendons, synovium, eye, heart, and vascular smooth muscle cells. It is implicated in ECM formation, structure, and cell signaling, and mutations in the *COMP* gene are known to cause skeletal diseases [36]. Interestingly, its upregulation has been shown to increase chondrocyte migration and collagen secretion. It also upregulates ECM stabilization via various mechanisms, such as collagen assembly and ECM signaling events. This is upregulated by mechanical loading and *TGFB1*, while it is inhibited by IL-1B [36]. Downregulation of this gene which stabilizes the ECM may support the hypothesis that this mechanism promotes microinvasion and subsequent metastasis. Furthermore, this finding aligns with the deactivation of several of the computationally predicted canonical pathways in IPA, including ECM organization, assembly of collagen fibrils and other multimeric structures, and integrin cell surface interactions. While minimizing structural constraints via ECM destabilization may facilitate micrometastasis, it also is possible that loss of stromal integrity, altered tumor purity, or variation in sample integrity may alter expression signals related to ECM stability of the samples collected.

#### 4.2.3. Collagen Type XIV Alpha 1 Chain Protein (*COL14A1*), Collagen Type XII Alpha 1 Chain (*COL12A1*)

Decreased expression of these collagen proteins in metastatic ES contributes to the predicted inhibition of the canonical pathways of ECM formation and assembly of collagen fibrils and other multimeric structures. Six of the seven series investigated *COL14A1* and *COL12A1*, and all samples within these series exhibited expression of these genes, with decreased expression in metastatic samples. Inhibition of *COL14A1* has been shown to have prognostic and diagnostic significance in some experimentally validated studies for other solid tumors [37,38]. This may be due to decreased levels of collagen leading to less anchorage-dependent growth [39], possibly promoting primary solid tumors to metastasize or grow through traditional tissue restraints. These findings suggest the expression of collagen proteins may additionally be associated with prognosis in metastatic ES.

### 4.3. Upstream Regulators

The top upregulated upstream regulator predicted via IPA, beta-estradiol, directly inhibits the second most affected upstream regulator, *TGFB1* (Figure 3B). Beta-estradiol also is predicted to upregulate several inflammatory markers, including tumor necorsis factor alpha (TNF-α) and interleukin 6 (IL-6), which in turn upregulate several of the top genes, including *SFTPB*, *SFTPC*, and *SLC6A14*. Beta-estradiol downregulates all but two of the top 10 downregulated genes, ubiquitin-specific peptidase 32 pseudogene 1 (*USP32A1*) and speedy/RINGO cell cycle regulator family member E15 (*SPDYE15*). In IPA’s proposed pathway, tumor protein 53 (TP53) is inhibited, and MYC proto-oncogene, bHLH transcription factor (MYC) and nuclear factor kappa B (NfKB) are activated. Beta-estradiol and several of its derivatives have been theorized to enhance malignancy proliferation and metastasis through several pathways, including the AHR/AKT/ERK1/2 pathway [40] and the calpain/YAP/beta-catenin pathway [41].

The *TGFB1* and dexamethasone pathways redemonstrate activation of anti-inflammatory markers, activation and inhibition of several top 10 genes, and inhibition of *TP53*. Note that these findings remain consistent as these pathways are inhibited.

### 4.4. Top Causal Networks

#### 4.4.1. JBI-802

JBI-802 is a lysine-specific demethylase 1 (LSD1) and histone deacetylase 6 (HDAC6) inhibitor that has shown significant potential in hematologic malignancies due to its antitumor and antiproliferative efficacy [42]. Downregulation of this causal network in metastatic ES samples indicates that addition of JBI-802 may lead to opposite genetic expression as what is seen in metastatic ES, thus suggesting it may be a promising therapeutic option. Here, IPA predicted that this inhibition subsequently activates mTOR, a key component of many tumorigenic pathways. JBI-802 inhibition also is predicted to increase expression levels of several genes already mentioned, including *SFTPB*, *SFTPC*, and *SCGB3A* (Figure 4A). Thus, utilizing JBI-802 as a potential therapeutic and its impact on downstream targets warrants further investigation.

#### 4.4.2. T-Box Transcription Factor 3 (*TBX3*)

*TBX3* represses E-cadherin in our projected pathways, which is thought to help epithelial-type malignancies undergo epithelial–mesenchymal transition and allow them to metastasize [43]. Our data supports the *TBX3* and E-cadherin inverse relationship, as integrin cell surface interactions are a top-inhibited canonical pathway (z-score −2.324). This is the role of *TBX3* in several musculoskeletal tumors such as osteosarcoma and chondrosarcoma [44,45]. Furthermore, downregulation of integrins has been shown to promote the epithelial–mesenchymal transition in prostate cancer cells, resulting in more aggressive phenotype [46].

Retinoic acid has shown to be an effective therapeutic in minimizing stem cell growth and differentiation by decreasing *TBX3* levels [47]. As one of the top causal networks for metastatic ES, this could be investigated as a therapeutic molecule. Another therapeutic approach could be with the 26-base guanine-rich DNA oligonucleotide aptamer to nucleolin, AS1411. TBX relies on nucleolin to create its tumorigenic effects by suppressing the tumor suppressor genes cyclin dependent kinase inhibitor 1A (*CDKN1A*) and 2A (*CDKN2A*) [48]. *TBX3* is suggested to be both a substrate and effector in the notable PI3K/AKT pathway, which is regarded as tumorigenic in multiple cancers, including melanoma [49]. *TBX3* also is theorized to be transcriptionally upregulated by c-Myc and post-transcriptionally upregulated by AKT, revealing another approach for therapeutic drugs; one study investigates targeting the c-Myc/AKT1/*TBX3* to treat rhabdomyosarcoma [50]. This aligns with our research results as AKT is estimated to be upregulated (*p*-value of 1.91 × 10^−5^).

#### 4.4.3. Cytochrome P450 Family 2 Subfamily B Member 6 (*CYP2B6*)

*CYP2B6* is one of the main enzymes responsible for activating the prodrugs cyclophosphamide and ifosfamide [51]. These two drugs are important components of the standard chemotherapeutic treatment for ES [52]. This upregulated causal network may explain how these drugs are either effective or not against ES metastasis.

### 4.5. Limitations

While this method of analysis produces robust information highlighting the potential pathogenesis, biomarkers, and therapeutic targets in metastatic versus primary ES, it is not without limitations. Utilizing the publicly available, de-identified database STARGEO to source samples limits the demographic and sample-level data available for further analysis. Furthermore, the integration of different series analyzed using varying microarray chips may introduce batch effect variation into the genes included within the analysis; thus, it is possible that genes significant to understanding the nature of metastatic ES may be inadvertently excluded from the study. Furthermore, to garner an appropriate sample size, metastatic tumors from multiple anatomic sites were utilized and pooled together. This may introduce heterogeneity due to varying body locations not attributable to the distinction between metastatic and primary tumors. This analysis serves primarily as a hypothesis-generating study to identify potential targets which may contribute to the pathogenesis of metastatic ES; thus, the genes and pathways identified herein warrant further investigation and validation to elucidate the definitive mechanism underlying their involvement in metastatic ES.

## 5. Conclusions

This study identified multiple potential biomarkers and therapeutic targets based on the pathway-level computational analysis and observed expression changes. Several observations also can be highlighted to characterize the nature of this malignancy. Three of the top ten upregulated genes are lung surfactant proteins: *SFTA3*, *SFTPC*, and *SFTPB*, which are heavily implicated in the healthy lung microenvironment and may provide insight into why the lungs are the top site of ES metastasis. Pro-*SFTPB* is a biomarker for other cancers and shows potential to be used as a biomarker for both metastatic ES and the progression of the disease during the treatment course. *SLC6A14* is a potential therapeutic target, as inhibition is suggested to decrease cancer growth in other studies. One of the most downregulated genes, *CXCL14*, is implicated in the AKT/ERK/mTOR pathway, and both this gene and the pathway offer therapeutic potential. Several of the most downregulated genes and the causal network of *TBX3* contribute to the predicted inhibition of canonical pathways, including ECM organization, assembly of collagen fibrils and other multimeric structures, and integrin cell surface interactions. These findings may contribute to the mechanistic hypothesis that decreasing structure enables microinvasion and metastasis. Further studies are required to validate the findings herein.

There are several therapeutic targets for one of the top causal networks, *TBX3*, including retinoic acid and AS1411. Another top causal network, *CYP2B6*, affects the metabolism of several key chemotherapeutic drugs, which may impact their effectiveness, or lack thereof, during treatment. Many gene targets and mechanistic pathways have been identified herein that may contribute to the pathogenesis of metastatic ES, serve as potential biomarkers identifying metastatic ES, or pose intriguing therapeutic options. Further research should prioritize validation of these findings in metastatic and primary ES.

## Figures and Tables

**Table 1:** Characteristics of samples included for analysis.

Series Accession Number	Case Sample Details	Control Sample Details	Excluded	Reference
GSE12102	7 ES metastasis samples (4 lung, 3 bone)	13 primary ES tumor samples (unknown location)	ES relapse tumor samples	[11]
GSE17618	4 ES metastasis samples (unknown location)	24 primary ES tumor samples	Normal muscle tissue samples, PNET samples, cell lines	[12]
GSE17674	4 ES metastasis samples (unknown location)	24 primary ES tumor samples	Normal muscle tissue samples, PNET samples, cell lines	[12]
GSE8303	2 ES metastatic samples (vertebrae and soft tissue)	10 primary ES tumor samples (3 femur, 1 soft tissue, 1 vertebrae, 1 rib, 1 clavicle, 1 radius, 1 pelvis)	Recurrent ES samples	[13]
GSE45544	4 ES metastatic samples (all lung)	5 primary ES tumor samples (2 rib, 1 perirenal, 1 femur, 1 Os ileum)	ES cell lines, ES relapse	[14]
GSE218758	14 metastatic ES samples (unknown location)	3 primary ES tumor samples (unknown location)	Non-primary and non-metastatic samples	[15]
GSE73166	2 metastatic ES samples (1 lung, 1 unknown location)	3 primary ES tumor samples (1 mandible, 1 temporal bone, 1 vertebra)	Osteosarcoma samples	Not available

**Table 2:** Top 10 upregulated and downregulated genes in metastatic ES versus primary tumors. The experimental log ratio indicates the magnitude and direction of change from primary ES on a natural logarithmic scale.

Upregulated Genes	Experimental Log Ratio	FDR-Corrected *p*-Value	Downregulated Genes	Experimental Log Ratio	FDR-Corrected *p*-Value
*ZNG1E*	0.675	2.54 × 10^−5^ (7.65 × 10^−4^)	*SPDYE15*	−0.677	3.36 × 10^−11^ (1.88 × 10^−8^)
*SFTA3*	0.582	4.48 × 10^−5^ (1.17 × 10^−3^)	*OGN*	−0.410	2.14 × 10^−3^ (2.01 × 10^−2^)
*SFTPC*	0.551	1.03 × 10^−2^ (6.06 × 10^−2^)	*CXCL14*	−0.393	1.22 × 10^−6^ (7.71 × 10^−5^)
*SFTPB*	0.511	1.12 × 10^−2^ (6.43 × 10^−2^)	*COMP*	−0.392	1.26 × 10^−7^ (1.34 × 10^−5^)
*SNORD114-2*	0.473	8.90 × 10^−3^ (6.87 × 10^−1^)	*USP32P1*	−0.320	4.03 × 10^−2^ (1.54 × 10^−1^)
*SNORD113-4*	0.467	1.26 × 10^−2^ (6.90 × 10^−2^)	*ENPP1*	−0.309	2.50 × 10^−5^ (7.59 × 10^−4^)
*ADH1B*	0.443	8.06 × 10^−4^ (9.89 × 10^−3^)	*COL14A1*	−0.306	1.68 × 10^−4^ (3.11 × 10^−3^)
*SNORD113-3*	0.435	1.42 × 10^−2^ (7.52 × 10^−2^)	*MYL1*	−0.301	5.74 × 10^−6^ (2.64 × 10^−4^)
*SLC6A14*	0.433	8.82 × 10^−6^ (3.62 × 10^−4^)	*COL12A1*	−0.298	1.11 × 10^−3^ (1.25 × 10^−2^)
*SCGB3A2*	0.416	1.12 × 10^−2^ (6.42 × 10^−2^)	*SULF1*	−0.290	2.72 × 10^0^ (1.18 × 10^−1^)

## Data Availability

Datasets utilized are available via NCBI’s GEO or https://www.ncbi.nlm.nih.gov/geo/, (accessed on 19 March 2026).

## Supplementary Materials

The following supporting information can be downloaded at: https://www.mdpi.com/article/10.3390/life16060901/s1, Table S1: Full List of Differentially Expressed Genes, Table S2: Full List of Differentially Expressed Genes Following FDR Correction.

## Abbreviations

The following abbreviations are used in this manuscript:

ES	Ewing sarcoma
EWS	Ewing sarcoma break point region 1
FLI1	Friend leukemia virus integration 1
ERG	ETS transcription factor ERG
EWSR1	Ewing sarcoma breakpoint region 1
NCBI	National Center Biotechnology Information
GEO	Gene Expression Omnibus
STAR	Search Tag Analyze Resource
IPA	Ingenuity Pathway Analysis
*COL12A1*	Collagen type XII alpha 1 chain
*COMP*	Cartilage oligomeric matrix protein
ECM	Extracellular Matri
*TGFB1*	Transforming growth factor beta 1
*TBX3*	T-box transcription factor 3
*ERBB2*	Erb-b2 receptor tyrosine kinase
*CYP2B6*	Cytochrome P450 family 2 subfamily B member 6
JARID2	Jumonji and AT-rich interaction domain containing 2
KDM1A	Lysine demethylase 1A
DNA	Deoxyribonucleic acid
RNA	Ribonucleic acid
IGF-1R	Insulin like growth factor 1 receptor
*CXCR4*	C-X-C motif chemokine receptor 4
SCGB	Secretoglobin
*ZNG1E*	Zn-Regulated GTPase Metalloprotein Activator 1E
*NERS*	Non-ETS-fused round cell sarcomas
*SFTA3*	Surfactant-associated protein 3
*SFTPC*	Surfactant protein C
*SFTPB*	Surfactant protein B
IL-1B	Interleukin 1 B
IL-23	Interleukin 23
*SLC6A14*	Solute carrier family 6 member 14
*SCGB3A2*	Secretoglobin family 3A member 2
TTF-1	Thyroid transcription factor 1
NKX2-1	NK2 homeobox 1
mRNA	Messenger RNA
C-X-C	Chemokine
*CXCL14*	C-X-C motif chemokine ligand 14
*COL14A1*	Collagen type XIV alpha 1 chain
*COL12A1*	Collagen type II alpha 1 chain
TNF-α	Tumor necrosis factor alpha
IL-6	Interleukin 6
*USP32P1*	Ubiquitin specific peptidase 32 pseudogene 1
*SPDYE15*	Speedy/RINGO cell cycle regulator family member E15
TP53	Tumor protein 53
MYC	MYC proto-oncogene, bHLH transcription factor
LSD1	Lysine-specific demethylase 1
HDAC6	Histone deacetylase 6
*CDKN1A*	Cyclin dependent kinase 1A
*CDKN2A*	Cyclin dependent kinase 2A
AKT	AKT serine/threonine kinase 1
